# Assessment of sexual dysfunction in patients with multiple sclerosis: a perspective from neurologist

**DOI:** 10.1186/s12883-022-02884-y

**Published:** 2022-09-29

**Authors:** Mariana Gaviria- Carrillo, Silvia Juliana Bueno-Florez, Paola Andrea Ortiz-Salas

**Affiliations:** 1 Universidad del Rosario, School of Medicine and Health Sciences, Neuroscience Center Neurovitae-UR, Neuroscience Research Group NEUROS, Bogotá, Colombia; 2 Neurologia, Fundacion Cardio Infantil Instituto de Cardiologia, Universidad del Rosario, Calle 163A # 13B – 60, Bogota, Colombia

**Keywords:** Sexual dysfunction, Multiple sclerosis, Neurologist, Barriers, Surveys and questionnaires

## Abstract

**Background:**

Sexual dysfunction (SD) is a common comorbidity in people with multiple sclerosis (pwMS). It affects the quality of life and remains an overlooked condition. The objective of this study was to describe how Colombian neurologists assess and treat SD and explore the barriers during sexual function evaluation.

**Methods:**

In this observational cross-sectional study we developed a questionnaire for neurologists with 4 sections (demographic data, evaluation and treatment of SD, and possible reasons for not discussing sexual dysfunction.) It was sent via email to 326 Colombian neurologists. We grouped the answers according to the type of consultation (neurologists from a MS program or no MS program). We described through absolute frequencies and proportions.

**Results:**

Fifty neurologists answered the survey. 64% stated that they usually study sexual dysfunction in neurological disorders. The main methods employed were private reading (86%) and attending conferences (14%). 5/50 participants have never attend pwMS; the Sect. 2–4 was not answered by them. 29% work in a MS program, all of them asked their patients about sexual function, but 18.75% of physicians working outside an MS program have never asked about it. Main reasons for not talking about sexual dysfunction were lack of knowledge (65.1%), presence of a companion (65.1%) and lack of time (55.8%). 91% of the neurologists reported that their patients usually and frequently ask about sexual function. Neurologists use informal questions to assess sexual function (80%), although 64.4% said that they are aware of SD questionnaires. When sexual dysfunction is detected, 91% of neurologists refer patients to another specialist and 87% do not start any treatment.

**Conclusions:**

Colombian neurologists are concerned with sexual function in pwMS, however it remains an underdiagnosed an undertreated condition. It is necessary to strengthen knowledge about the diagnosis and treatment of sexual dysfunction in pwMS, for neurologists and patients. It is also imperative to eliminate barriers around the topic and include sexual function evaluation and treatment as the routine care of pwMS.

## Highlights

• Sexual dysfunction among people with multiple sclerosis (pwMS) is frequent and has an impact on quality of life.

• Neurologists are concerned about sexual function in pwMS.

• Patients usually report sexual dysfunction to neurologists.

• There is still a gap in the treatment and evaluation of sexual dysfunction in pwMS.

## Introduction

Multiple sclerosis (MS) is a chronic, inflammatory and degenerative disease that mostly affects young adults [1] and it is a common cause of neurological disability [2]. In addition to the typical MS symptoms, these subjects may also experience sexual dysfunction (SD), which greatly affects mental, emotional, and physical health as well as having major impact on quality of life [3]. Nowadays, the prevalence of SD in subjects with MS ranges between 40–80% for women and 50- 90% for men [4]. MS can affect sexual function in several different ways. Foley et al. proposed a conceptual model to categorize SD into three main groups [5]. Primary causes of SD are demyelinating lesions and axonal loss among neural pathways in the spinal cord and brain, which control sexual function. Secondary causes of SD are neurological symptoms and physical disabilities that impact sexual function, such as fatigue, tremor, ataxia, spasms, or incontinence. Tertiary causes of SD are related to emotional and psychosocial concerns caused by MS [3, 5].

In spite of having a high prevalence and a significant impact on quality of life [6], SD is rarely screened which leads to underdiagnoses and delayed or absent treatments [7, 8]. Similarly, patients have reported that physicians do not ask about sexual problems [8, 9]. Furthermore, some data reported that 63% of patients have never talked to physicians or healthcare providers about their sexual function [10]. Patients and physicians have identified barriers such as: Apprehension of physicians to ask patients about their sexual function or even bring up the subject of sex [11], lack of time during a consultation, presence of a family member or friend during the consult, or a lack of knowledge of this comorbidity [9, 12].

Considering the sizable impact SD has on quality of life, self-image, self-esteem and interpersonal relationships, sexual function needs to be addressed and included as a part of the comprehensive care of patients with MS [10]. Identifying how neurologists asses and treat SD and the barriers that physicians face during evaluation, is paramount in order to improve and optimize care, as well as sexual function education among neurologists. Therefore the objective of this study is to describe how Colombian neurologists assess and treat SD and explore the barriers during sexual function evaluation.

## Methods

This was an observational cross-sectional study, carried out in September 2020, in which a survey was sent via e-mail to 326 Colombian neurologists, members of the Colombian Association of Neurology. We asked for permission to the Colombian Association of Neurology committee to send the survey, every participant provided consent to answer the survey. The questionnaire was designed and presented in Spanish and contained 20 questions divided into 4 sections. The first one explored demographic data (age, years working as a neurologist, city where they work, and if they belong to an MS program or not). The second section investigated how neurologists evaluate SD (if they know any tool or scale for their assessment and which one, they apply in clinical practice). A third section asked the neurologists about their management of cases when sexual dysfunction is detected. And, finally, the last section inquired into possible barriers for assessing sexual function.

We analyzed the neurologist’s answers according the type of consultation, if they belong to a multiple sclerosis program, we grouped them as: MS program, otherwise if them do not work inside a multiple sclerosis program, we classified them as no MS program.

A database was created in Excel version 2020, which included the patient record, personal data, and the variables of the questionnaire. The information was processed using SPSS version 22 statistical packages. For the description of qualitative variables, frequency distributions and percentage distributions were used. For quantitative variables, measures of central tendency such as average and measures of dispersion such as standard deviation were used. The Colombian Association of Neurology gave permission to send the survey to its members and survey data were anonymous. The datasets generated and/or analyzed during the current study are available in the OSF repository (https://osf.io/e2sc7/?view_only=a0f470c53eb04a49baa1516df1717d0c).

## Results

### Demographic characteristics

50 neurologists answered the survey. Demographics are summarized in Table 1. We explored how frequently neurologists study sexual dysfunction in neurological disorders. Amongst respondents, 64% stated that they usually study sexual dysfunction in neurological disorders. The main methods employed were private reading (86%) and attending conferences (14%).

**Table 1:** Demographic characteristics

	N (%)
Age (years)	
30–40	22 (44)
41–50	11(22)
51–60	13(26)
> 60	4(8)
Years caring for patients as neurologist	
< 5	19 (38)
6–10	3(6)
> 10	28(56)
How frequently do you treat pwMS?	
Occasionally treat MS patients	21 (47)
Usually treat MS patients	11 (24)
Treat MS patients in a MS program	13(29)

From our sample, 5/50 have never attend pwMS, so the section regarding sexual function in MS (Sect. 2–4) was not answered by them. From the 45 neurologists who attend pwMS, 13 (29%) work in a MS program and 32 (71%) attend pwMS outside a MS program.

### Perspectives, assessment, and management of sexual dysfunction

The following sections (Perspectives, assessment, and management of sexual dysfunction and reasons for not discussing sexual dysfunction in MS patients) included only subjects who attends pwMS (*n* = 45). All neurologists from an MS program ask their patients about sexual function; however, 18.75% of physicians working outside an MS program have never asked about it. In addition, 91.1% of the neurologists reported that their patients usually and frequently ask about sexual function.

Neurologists normally use informal questions to assess sexual function (80%), although 64.4% said that they are aware of SD questionnaires.

Tools for the assessment of sexual dysfunction are used more frequently by neurologists who belong to an MS program, center, or clinic compared to neurologists who work at a regular healthcare facility (38% v. 12%).

The tools most commonly used included the MSISQ 19 (Multiple Sclerosis Intimacy and Sexuality Questionnaire-19) MSISQ 15. Others tools, used only by three neurologist were SEA-MS-F (Sexual Dysfunction Management and Expectations) and FSFI (Female Sexual Function Index).

When sexual dysfunction is detected, 91% of neurologists refer patients to another specialist (urologist or gynecologist). Most of them (87%) do not start any treatment. Some managements started by the neurologists are: lubricating gel, phosphodiesterase-5 inhibitors (PDE5) and sertraline.

Regarding tests for evaluation of SD, 20% requested it (dorsal magnetic resonance imaging, pudendal nerve conduction or a hormonal profile).

All the neurologists consider sexual dysfunction in pwMS to be an important condition.

### Reasons for not discussing sexual dysfunction in MS patients

The reasons for not discussing sexual dysfunction in pwMS were reported in Table 2.

**Table 2:** Perception of sexual dysfunction

Reasons for not discussing sexual dysfunction in pwMS	*N* = 45 (%)
Lack of time	24 (55.8)
Lack of knowledge	28 (65.1)
Patients bring a companion to consultation	28 (65.1)
Discussing about sexual dysfunction generates anxiety and discomfort	6 (14)
Not enough skill to manage those topics and therefore feels uncomfortable	7 (16.3)
Treating sexual dysfunction is not part of a neurologist’s role	1 (2.3)
Sexual dysfunction is not related to MS	0
Cultural or religious factors	6(14)

## Discussion

Colombian neurologists are concerned with sexual function in pwMS, however it remains an underdiagnosed condition and it poses a special challenge for neurologists working outside an MS program. Our results are somewhat similar to previous data, nonetheless, there has been a tendency towards better diagnoses of SD in MS. Previous studies from 1989 reported that pwMS had never discussed SD with their medical practitioner [13]. In 2003, a survey of health care providers on SD in MS found that most of them are concerned about sexual problems, however only a minority investigated them [14]. More recent studies have reported that about 15%–55% of neurologists discussed this topic with patients [8, 15–17].

Possible explanations for these heterogeneous results include differences among design methods (studies were carried out in different centers, and countries with different levels of specialization, participants are not homogenous; some of them are general neurologists and others are neurologists specialized in MS) and perception changes over time.

The fact that sexual function remains underdiagnosed can be explained by some barriers reported by both neurologists and patients. The main barriers for SD evaluation found in this survey were lack of time, lack of knowledge, or the presence of a companion during the consultation. Previous studies have identified similar barriers perceived by health care professionals (HCP) such as lack of time, lack of knowledge, or the presence of a companion during the consultation [9, 18]. Additionally, HCPs are concerned because they are not sure if patients want to be asked about sexual function, and some HCPs think this topic may be invasive [17]. In contrast, most patients perceive a need to talk about their sexual function [17]. Further, in our sample neurologists usually explore SD using informal questions; however, more than half know that sexual dysfunction questionnaires for MS exist. This conduct can be explained by the lack of time available during the consultation or lack of training on how to apply and interpret these questionnaires.

Although the majority of Colombian neurologists ask about SD, only 13% initiate some treatment and most of them refer the patient to another specialist. This is in line with previous studies that reported that treatment satisfaction for SD was generally poor [8].

Similarly, Redelman reported that more than half of patients with SD did not obtain help for their issue [16]. This reflects that it is not only important to ask and diagnose, but also to treat SD in these patients. Some problems related to sexual dysfunction can be treated by a neurologist. For example, secondary causes of SD could be caused by spasms or neuropathic pain. These symptoms can be managed with carbamazepine and pregabalin respectively [3]. Moreover, pwMS can have self-esteem issues and perceive that their body is less attractive or desirable due to subcutaneous injections of interferons or glatiramer. Thus, a change to an oral disease-modifying therapy might be helpful.

According to the gaps identified in this survey and based on a narrative review of the literature [9], we suggest possible recommendations to eliminate gaps and barriers. First of all, the lack of application of questionnaires such as MSISQ 19 or 15 could be associated with the short times available for consultations. These tools not only help to identify the presence of SD, but they also categorize as primary, secondary or tertiary causes of SD. This is important when choosing an appropriate treatment and follow-up. Many of these tools can be self-administered by pwMS, so we recommend that they could be filled out in the waiting room, or the questionnaire could be sent beforehand via email, completed at home, and brought to the neurologist. However, a previous study reported that patients preferred health care professionals to explore sexual function by direct questions in their homes [17]. Therefore, it might be important that neurologists explore with their patients how they want their sexual function to be assessed. Moreover, including SD topics in conferences, symposia and local education sessions addressed by neurological associations or MS programs could be a plausible and useful strategy to strengthen sexual function knowledge. Additionally, the evaluation of patients within a demyelinating disease program can strengthen comprehensive evaluation, since there is constant updating among team members with different subspecialties that make it possible to identify different symptoms and, therefore, enable an early referral to other specialists.

Among the limitations of this study is that the survey was only answered by 15.3% of the members of the Colombian Association of Neurology. Likewise, the design of the survey is concise and may fall short in evaluating SD in pwMS comprehensively. Our survey only provides a broad landscape of the perceptions of patients and the approach to evaluate and manage sexual dysfunction in patients with multiple sclerosis. However, we managed to make a general description of approaches to sexual dysfunction symptoms and their possible limitations during the evaluation. This study opens the possibility of continuing to research the different symptoms that pwMS present and that impact their quality of life, and the importance of multidisciplinary management.

## Conclusions

Sexual dysfunction is still an unmet need in subjects with multiple sclerosis. It is necessary to strengthen knowledge about the diagnosis and treatment of sexual dysfunction in pwMS, for neurologists and patients. It is also imperative to eliminate barriers around the topic and include sexual function evaluation and treatment as the routine care of pwMS.

## Data Availability

Data is publicly accessible through OSF repository https://osf.io/n4vxj/quickfiles.
